# Modulation of Orthodontic Tooth Movement by Statins: A Systematic Review of Animal Studies

**DOI:** 10.3390/dj14060331

**Published:** 2026-06-01

**Authors:** Roberta Crispino, Francesca Zara, Massimiliano Vella, Lara Colaianni, Cinzia Maspero, Marco Serafin, Alberto Caprioglio

**Affiliations:** 1 Department of Biomedical Surgical and Dental Sciences, University of Milan, 20122 Milan, Italylara.cola2000@gmail.com (L.C.); marco.serafin@unimi.it (M.S.);; 2 Fondazione IRCCS Ca’ Granda Ospedale Maggiore Policlinico Istituto di Ricovero e Cura a Carattere Scientifico di natura pubblica via Francesco Sforza, 28, 20122 Milan, Italy

**Keywords:** statins, orthodontic tooth movement, orthodontic relapse, bone remodeling, pharmacological administration

## Abstract

**Objective:** This systematic review aims to evaluate the effects of statin administration on orthodontic tooth movement (OTM) and post-treatment relapse in animal models. **Materials and Methods:** Following PRISMA guidelines and PROSPERO registration (CRD42025612449), a comprehensive literature search was performed in PubMed, Scopus, and the Cochrane Library up to December 2025. The search strategy included studies on animal models treated with any form of statin during OTM or relapse phases. Eligibility criteria were defined using the PICOS framework. Data extraction focused on study characteristics, statin administration protocol, orthodontic mechanics, and both histological and molecular markers of bone remodeling. Risk of bias was assessed with SYRCLE’s tool. **Results:** Seven in vivo animal studies met the inclusion criteria. Simvastatin and atorvastatin were investigated using heterogeneous experimental models, doses, administration routes, orthodontic mechanics, and follow-up periods. Findings suggested that statins may reduce active orthodontic tooth movement or post-orthodontic relapse in some experimental settings, but effects were not uniform across studies. Histological outcomes, when reported, generally indicated reduced osteoclast activity, fewer resorption lacunae, or more mature alveolar bone in statin-treated animals. Molecular outcomes were less consistently reported and mainly involved OPG/RANKL-related pathways, Runx2, or ALP expression. **Conclusions:** Statins, particularly simvastatin, show potential to modulate orthodontic tooth movement and reduce relapse by influencing bone metabolism. Nevertheless, due to methodological variability and the exclusive reliance on animal models, these results cannot yet be translated into clinical recommendations. Current preclinical evidence suggests that statins may modulate bone remodeling during active orthodontic tooth movement and after appliance removal. However, the evidence remains limited to a small number of heterogeneous animal studies with incomplete reporting of key outcomes and several unclear risk-of-bias domains. Clinical translation is premature, and more standardized preclinical studies are required before human investigations can be justified.

## 1. Introduction

Orthodontic tooth movement (OTM) results from the controlled application of mechanical forces, initiating a complex mechanotransduction process, whereby the physical force applied to teeth is translated into biochemical signals within the periodontal ligament and surrounding alveolar bone. Compression and tension zones develop specific micro-environmental conditions: in compressed regions there is reduced vascularity, promoting hypoxic conditions that stimulate osteoclast differentiation and activity, facilitating bone resorption. Conversely, in tension regions, increased vascular perfusion enhances osteoblastic activity and bone apposition, remodeling the alveolar bone to accommodate tooth movement [1,2,3].

The molecular pathway underlying OTM is intricate, involving various signaling molecules that orchestrate localized tissue remodeling. These signals are responsible for the synthesis of pro-inflammatory mediators, such as prostaglandins and leukotrienes, which play critical roles in regulating osteoblastic and osteoclastic activities. In parallel, cells within the periodontal ligament, including osteoblasts, fibroblasts, and resident immune cells, release an array of cytokines which further amplify the inflammatory cascade and potentiate cellular differentiation necessary for bone remodeling [4,5].

Importantly, each step in these complex pathways offers potential points of pharmacological intervention, where endogenous or exogenous agents might modulate the OTM. For instance, systemic drugs have been shown to influence it by either accelerating or inhibiting the rate of movement [6,7]. Therefore, the impact of pharmacodynamics on orthodontic treatment outcomes, as drugs prescribed for conditions unrelated to orthodontics, can inadvertently alter the efficacy and rate of OTM.

Statins, a widely prescribed class of lipid-lowering drugs, exert a well-documented influence on bone metabolism. As competitive inhibitors of 3-hydroxy-3-methylglutaryl-coenzyme A reductase, statins inhibit the mevalonate pathway, reducing cholesterol biosynthesis and yielding a secondary pleiotropic effect on bone tissue. Specifically, statins have been found to upregulate bone morphogenetic protein-2, enhance osteoblast survival by inhibiting apoptosis, and disrupt osteoclastogenesis by modulating of the OPG/RANKL/RANK pathway [8,9]. These biochemical effects collectively promote bone formation and decrease its resorption, suggesting a potential therapeutic role for statins in modifying the response of osseous tissue during and after orthodontic interventions [9].

Given this background, there is a compelling rationale hypothesizing that statins may influence the OTM by altering bone remodeling dynamics, thus potentially reducing the magnitude of orthodontic relapse. However, the exact impact of statins on the cellular and molecular mechanisms of OTM remains insufficiently understood and mainly limited to animal studies. Because active orthodontic tooth movement and post-treatment relapse occur under different biomechanical and biological conditions, these outcomes were considered separately throughout the review. This systematic review aimed to evaluate, in animal models, whether statin administration influences two distinct orthodontic outcomes: active orthodontic tooth movement during force application and post-orthodontic relapse after appliance removal. Secondary objectives were to summarize the histological and molecular evidence on statin-related modulation of alveolar bone remodeling, with particular attention to osteoclast activity, bone formation, and OPG/RANKL-related pathways.

## 2. Materials and Methods

**Figure 1:**
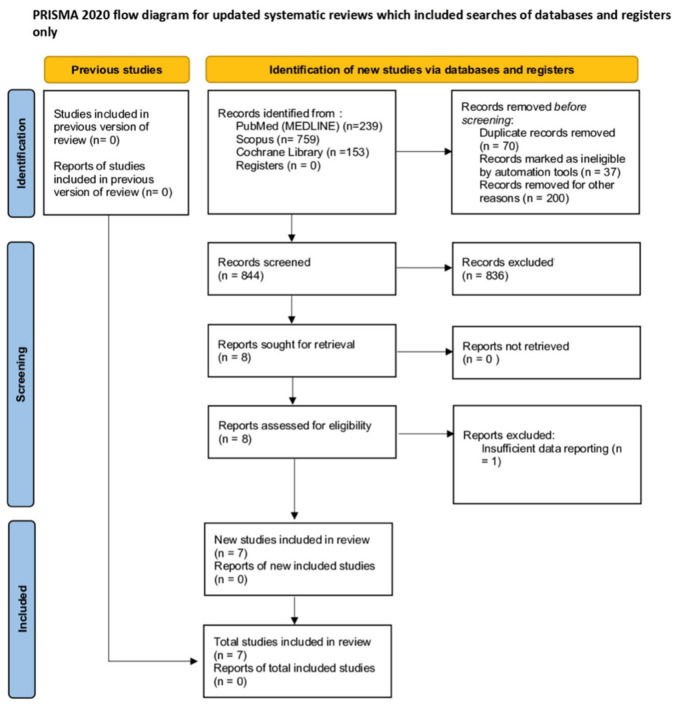
PRISMA 2020 flow diagram for updated systematic reviews which included searches of databases and registers only.

The present systematic review was conducted in adherence to the PRISMA checklist (Preferred Reporting Items for Systematic Reviews and Meta-Analyses) guidelines and was registered in the International Prospective Register of Systematic Reviews (PROSPERO), registration number CRD42025612449 [10] (Figure 1) (Supplementary Materials File S1).

A comprehensive literature search was conducted in PubMed (MEDLINE), Scopus, and the Cochrane Library databases. The search spanned the database inception dates to December 2025. The full electronic search strategy is available in Supplementary Table S1.

Studies were selected based on PICOS criteria [11]:

Population (P): In vivo animal models undergoing experimentally induced orthodontic tooth movement and/or post-orthodontic relapse after appliance removal.

Intervention (I): Administration of any statin or HMG-CoA reductase inhibitor, including but not limited to simvastatin, atorvastatin, pravastatin, rosuvastatin, fluvastatin, lovastatin, or pitavastatin, irrespective of dose, formulation, timing, or route of administration.

Comparison (C): Placebo, vehicle, no-treatment control, or contralateral/internal control groups, when applicable.

Outcomes (O): Primary outcomes were active orthodontic tooth movement, expressed as rate or magnitude of tooth displacement during force application, and post-orthodontic relapse, expressed as linear displacement or percentage relapse after appliance removal. Secondary outcomes included histological and microstructural parameters of bone remodeling, such as osteoclast number, resorption lacunae, bone volume, bone maturation, and periodontal ligament remodeling, as well as molecular markers including OPG, RANKL, OPG/RANKL ratio, Runx2, ALP, and other reported markers of osteogenesis or osteoclastogenesis.

Study design (S): Controlled in vivo animal experimental studies.

The formulated research question based on this PICOS was the following: In in vivo animal models (P), does the administration of statins or HMG-CoA reductase inhibitors (I), compared with vehicle, placebo, no treatment, or internal controls (C), affect orthodontic tooth movement, post-orthodontic relapse, and related histological or molecular markers of alveolar bone remodeling (O)?

Two reviewers (R.C. and F.Z.) independently screened titles and abstracts. Full-text versions were retrieved for studies meeting the eligibility criteria or when eligibility was uncertain. Reports that did not satisfy the inclusion criteria, such as studies including human populations, were excluded during the screening process. Discrepancies were discussed and resolved by consensus or through the involvement of a third reviewer (M.S.) if necessary. Agreement between reviewers was calculated using Cohen’s kappa coefficient (0.94) to assess the reliability of the selection process too [12].

A standardized form was developed to extract detailed study information. Data extraction included the following (Table 1):

- Study Characteristics: Authors, year of publication, animal species, and sample size.
- Intervention Details: Type of statin, dosage, route of administration, and duration of treatment.
- Orthodontic Protocol: Appliance type, applied force magnitude, and force duration.
- Outcome Measures: Primary (tooth movement measurements such as amount and rate as well as relapse after OTM) and secondary (histological findings, including osteoclast/osteoblast counts and sites of bone remodeling, molecular markers and their expression levels).
- Study Findings: Reported effects of statins on OTM and orthodontic relapse.

Similarly to the selection process, the same two reviewers independently extracted the data, and cross-verification was conducted to ensure consistency.

The risk of bias (ROB) was evaluated using SYRCLE’s Risk of Bias tool for animal studies, designed to assess methodological quality across domains specific to animal research [13]. Each ROB was scored as “low risk,” “high risk,” or “unclear risk” based on study descriptions. Discrepancies in scoring were resolved through discussion (Table 2). A meta-analysis was not performed because the included studies showed substantial methodological and biological heterogeneity. Differences in animal species, statin type and dosage, route of administration, orthodontic mechanics, outcome definitions, and follow-up periods prevented a reliable quantitative synthesis of the data.

**Table 1:** Summary of the main characteristics of the selected studies.

Study	Animal Model	Statin Administration	Route	Force/ Duration	OTM	Relapse	Histological Findings	Molecular Findings	Main Outcome
Han G, 2010 [14]	Wistar rats	2.5 mg/kg/die simvastatin	Intraperitoneal injections	50 cN/21 d	NR	↓ relapse	↓ RP, smoother bone surface	↑ OPG/RANKL	Reduced relapse
Vieira GM, 2015 [15]	Wistar rats	5 mg/kg simvastatin	Oral gavage	75 cN/18 d	NR	NR	NR	NR	No significant inhibition
Dolci GS, 2017 [16]	Wistar rats	15 mg/kg atorvastatine	Oral gavage	50 cN/21 d	NR	↓ relapse	↓ OC count	↑ OPG	Reduced relapse
AlSwafeeri H, 2018 [17]	White New Zealand rabbits	0.5 mg/480 µL simvastatin solution	Local injections	100 cN/21 d	No difference	No difference	↑ bone maturation	NR	Reduced resorption
Dolci GS, 2018 [18]	Wistar rats	15 mg/kg/day atorvastatine	Gavage	50 cN/21 d	↓ OTM	NR	↓ OC count	NR	Temporary inhibition
AlSwafeeri H, 2019 [19]	White New Zealand rabbits	0.5 mg/480 µL simvastatin solution	Local injections	100 cN/21 d	NR	↓ relapse	↓ RP and OC	NR	Reduced relapse
Liu X, 2022 [20]	Sprague Dawley rats	2 mg/500 µL simvastatin/exosomes	Local injections	49.5 cN/14 d	NR	↓ relapse	↑ bone maturation	↑ Runx2, ↑ ALP	Enhanced osteogenesis

CG: control group; TG: test group; OC: osteoclasts; FB: fibroblasts; RP: resorption pits; PDL: periodontal ligament; NR: not reported; * grams (g) of force were converted in centiNewtons (cN) with the following equation: 1 cN = 1.01 g; ^ T1: 7 days; T2: 14 days; T3: 21 days after appliance removal; ° T1: 7 days; T2: 14 days; T3: 21 days from baseline. ↓ decreased; ↑ increased.

**Table 2:** The risk of bias (ROB) evaluated using SYRCLE’s Risk of Bias tool for animal studies. Each ROB was scored as “low risk,” “high risk,” or “unclear risk”.

	Selection Bias			Performance Bias		Detection Bias		Attrition Bias	Reporting Bias	Other
Article	Sequence Generation	Baseline Characteristics	Allocation Concealment	Random Housing	Blinding	Random Outcome Assessment	Blinding	Incomplete Outcome Data	Selective Reporting	Other Sources of Bias
Han G, 2010 [14]	Unclear	Low risk	Unclear	Unclear	Unclear	Unclear	Low risk	Low risk	Low risk	Low risk
Vieira GM, 2015 [15]	High risk	Low risk	Unclear	Unclear	Unclear	Unclear	High risk	Low risk	High risk	Low risk
Dolci GS, 2017 [16]	Unclear	Low risk	Unclear	Unclear	Unclear	Unclear	Low risk	Low risk	Low risk	Low risk
AlSwafeeri H, 2018 [17]	Low risk	Low risk	Low risk	Low risk	Low risk	Low risk	Low risk	Low risk	Low risk	Low risk
Dolci GS, 2018 [18]	Unclear	Low risk	High risk	Unclear	Unclear	Unclear	High risk	Low risk	Low risk	Low risk
AlSwafeeri H, 2019 [19]	Low risk	Low risk	Low risk	Unclear	Low risk	Low risk	Low risk	Low risk	Low risk	Low risk
Liu X 2022 [20]	Unclear	Low risk	Unclear	Unclear	Unclear	Unclear	Low risk	Low risk	Low risk	Low risk

## 3. Results

A total of 1151 articles were retrieved from the systematic search across PubMed, Scopus, and the Cochrane Library. After removing duplicates and articles not relevant to the topic, 844 articles remained. During the screening process of titles and abstracts against eligibility criteria, eight studies were identified as potentially eligible for full-text review. Following a detailed assessment, one study was excluded due to insufficient data on statin administration relevant to OTM, resulting in a final inclusion of seven studies for qualitative analysis. Table 1 reports the qualitative analysis from the included studies.

The included studies applied similar protocols to induce orthodontic tooth movement but varied in terms of animal model, type of statin used, administration route, dosage, and outcome measures. Of the seven studies, five used male rats, while the remaining two utilized male rabbits [14,15,16,17,18,19,20]. Simvastatin was examined in five studies [14,15,17,19,20], while two studies focused on atorvastatin [16,18]. The primary methods of statin administration were local injection (intraligamentous or submucosal) and systemic routes (oral gavage or intraperitoneal injection). The results of individual studies are summarized in Table 1.

### 3.1. Effects of Statins on Orthodontic Tooth Movement

The studies collectively reported that statins impact bone metabolism and modulate OTM through mechanisms related to bone resorption and formation. In the two studies investigating simvastatin and OTM [17,18], heterogeneous findings were reported. In detail, Dolci et al. [18], who administered simvastatin by oral savage, observed a significant reduction in OTM rate and magnitude compared to controls. Al Swafeeri et al. [17], on the other hand, did not report significant effects on OTM and relapse after local injections of simvastatin. Both studies, however, consistently reported an inhibition of bone resorption compared to controls; additionally, from a histological perspective, an increase in bone maturation and a decrease in osteoclasts was observed after statin administration.

### 3.2. Orthodontic Relapse and Bone Remodeling

Five studies explored the effects of statins on orthodontic relapse following the removal of orthodontic force. The data on post-orthodontic relapse was reported either as a percentage (i.e., “relapse rate”) or as a linear distance. In all cases, only summary statistics—specifically, means and standard deviations—were provided, with no additional individual-level or distributional data available. Simvastatin was shown to significantly reduce relapse in three of these studies, attributed to enhanced alveolar bone formation and an increased OPG/RANKL ratio, leading to sustained bone density and structural integrity [14,19,20]. However, two studies reported no statistically significant differences in relapse magnitude between simvastatin and control groups [15,17]. Moreover, in a study on micro-CT radiograph images, simvastatin was proved not only to not reduce relapse but also to not alter bone mineral density at a low dosage [15]. Atorvastatin’s effect was examined by Dolci et al., who demonstrated that local injections decreased osteoclastogenesis and increased OPG expression, thus reducing post-orthodontic relapse [16]. The contrasting results among the different studies may reflect variability in dosage, administration routes, and the timing of statin administration relative to OTM and force removal.

### 3.3. Histological and Molecular Findings

Histological examination consistently showed that statins positively influenced bone microarchitecture, with decreased osteoclast presence and increased osteoblastic activity in tension regions. Molecular analysis revealed an elevated expression of OPG and reduced RANKL expression in statin-treated groups, indicating a shift towards bone preservation [14,16]. Although some studies using higher doses or specific delivery systems reported more pronounced changes in bone remodeling outcomes, dose, route of administration, formulation, animal species, and timing of administration varied simultaneously. Therefore, the current evidence does not allow a reliable assessment of dose–response or route-specific effects [17,18,19,20].

### 3.4. ROB Assessment

The risk of bias assessment highlighted methodological strengths and limitations across studies (Table 2). SYRCLE’s tool revealed important limitations in the reporting and conduct of the included animal studies. No overall certainty rating was assigned because a formal certainty-of-evidence framework was not applied. Although baseline characteristics, incomplete outcome data, and other sources of bias were generally judged as low risk in several studies, many domains were rated as unclear risk because methodological details were insufficiently reported. Sequence generation, allocation concealment, random housing, blinding of caregivers or investigators, random outcome assessment, and blinding of outcome assessment were frequently unclear. Some studies also showed high risk in specific domains, including sequence generation, allocation concealment, detection bias, or selective reporting. Therefore, the internal validity of the available evidence should be considered limited by incomplete reporting of key methodological domains, and the findings should be interpreted cautiously.

## 4. Discussion

The present systematic review showed the results from the screening of a limited number of animal studies. This systematic review identified a small body of in vivo animal evidence suggesting that statins may modulate alveolar bone remodeling during active orthodontic tooth movement and post-orthodontic relapse. However, the magnitude and direction of these effects were not consistent across studies, and the evidence was limited by heterogeneity in animal species, drug type, dose, route of administration, orthodontic mechanics, timing of administration, and outcome assessment. Therefore, the findings should be interpreted as preliminary and hypothesis-generating. However, effects on orthodontic relapse remain inconclusive, likely due to methodological variability and differential drug pharmacodynamics across studies. The large number of unclear-risk judgments should not be interpreted as absence of bias. Rather, it indicates incomplete reporting of key procedures required to protect internal validity in animal experimental studies. This limitation reduces confidence in the observed effects and prevents firm conclusions regarding the magnitude and reproducibility of statin-related modulation of OTM or relapse.

Statins represent a class of medications extensively used in the adult population [21,22]. In fact, due to their efficacy in lowering cholesterol levels, this class of medication is the world-wide first choice prescription against lipid disorders and is commonly administered in dyslipidemic adults at cardiovascular risk. A biological role of statins on bone turnover has been ascertained in previous research [8,9,23,24]. Statins function as competitive inhibitors of 3-hydroxy-3-methylglutaryl-coenzyme A (HMG-CoA) reductase; the direct effect of this inhibition is the interruption of the mevalonate pathway, eventually impairing hepatic cholesterol biosynthesis. Notably, however, the mevalonate pathway is involved not only in cholesterol metabolism but also in the production of diverse biomolecules; therefore, a pleiotropic effect of statins has been recorded [9,25]. In detail, these medications were observed to act as biomodulators of bone metabolism: indeed, statins were found to induce an upregulation of bone morphogenic protein-2 (BMP-2) in rodents [26], to inhibit osteoblast apoptosis and to prevent osteoclastogenesis by altering the OPG/RANKL/RANK pathway [8].

The results of the analyzed studies may be interpreted considering these biological mechanisms. Alterations of bone turnover and of the OPG/RANKL ratio microscopically, and of OTM and relapse macroscopically, can be expected after statin administration, especially in the event of a systemic delivery route. As a matter of fact, in all analyzed studies, there was agreement concerning the observed histologic effects of statins. Specifically, compared to controls, samples of subjects exposed to statins presented less bone resorption pits, more mature bone, smoother bone surfaces and a reduced osteoclast count.

Nonetheless, on the clinical side, the analyzed research papers retrieved conflicting results, as four studies [14,16,19,20] observed a reduction in orthodontic relapse, one study [18] noted a transient inhibition of osteoclastogenesis, and two studies [15,17] did not find any significant reduction in post-orthodontic relapse. Considerable heterogeneity among the included studies should, however, be emphasized, as differences were present in animal models, statin type, dosage, formulation, route and timing of administration, and outcome assessment. Consequently, direct comparisons between administration protocols should be interpreted with caution, and no definitive conclusions can be drawn regarding the superiority of local versus systemic administration or the existence of a dose-dependent effect. The most common delivery routes were oral gavage and local injections, while intraperitoneal injections were chosen only in one study. Notably, an innovative approach was proposed by Liu et al., who isolated and identified periodontal ligament stem cells (PDLSCs) and their exosomes (PDLSCs-Exo); in this experimental setting, simvastatin was encapsulated in exosomes and injected locally [20]. According to the authors’ observations, encapsulating simvastatin into the exosomes may improve simvastatin solubility and enhance the inhibition of relapse in rats. Regarding local injections, a statistically and clinically significant reduction in relapse was observed in two studies [19,20] out of three; on the other hand, statins delivered by oral gavage were shown to reduce relapse in one study [16], to have temporary effects in one study [18] and to not alter OTM in another two studies [15,17]. Lastly, inhibition of relapse was observed in the research setting using intraperitoneal injections. Overall, while several studies suggested a potential inhibitory effect of statins on orthodontic relapse, the variability in experimental designs and treatment protocols limits the possibility of establishing reliable conclusions regarding the influence of specific administration routes or dosages.

## 5. Limitations

This review has several limitations. First, the available evidence was limited and consisted exclusively of in vivo animal studies. Only seven studies met the eligibility criteria, and even fewer contributed to each specific outcome. Second, substantial heterogeneity was present across studies, including differences in animal species, statin type and dosage, route and timing of administration, orthodontic protocols, relapse observation periods, and outcome assessment methods. This variability prevented quantitative synthesis and limited direct comparison among studies. In addition, outcome reporting was often incomplete and inconsistent. Orthodontic tooth movement and post-orthodontic relapse were not always evaluated within the same studies and were reported using different measurement methods. Histological and molecular findings were also assessed using non-standardized markers and protocols, limiting comparability across investigations. Furthermore, the risk-of-bias assessment revealed several unclear-risk domains, mainly due to insufficient reporting of methodological procedures, reducing confidence in the internal validity of the included studies. Finally, although experimental evidence supports the biological plausibility of statin-mediated modulation of bone remodeling, the present findings cannot be directly extrapolated to clinical orthodontics. Differences between animal models and human orthodontic treatment, together with the lack of standardized preclinical protocols, make clinical translation premature. Future research should focus on standardized in vivo animal studies with consistent methodologies and outcome reporting, while complementary in vitro studies may help clarify the underlying cellular and molecular mechanisms.

## 6. Conclusions

The available in vivo animal evidence suggests that statins may influence alveolar bone remodeling during active orthodontic tooth movement and post-orthodontic relapse, mainly through mechanisms involving reduced osteoclast activity and modulation of osteogenic or osteoclastogenic pathways. However, the findings are heterogeneous, outcome reporting is incomplete, and several studies present unclear risk-of-bias domains. Evidence regarding relapse prevention and active OTM modulation remains suggestive but inconclusive. At present, no clinical recommendation can be made. Future animal studies should use transparent randomization and blinding procedures, predefined outcome measures, comparable force systems, standardized relapse models, clearly reported statin dose and formulation, and consistent histological and molecular endpoints. Only after reproducible preclinical evidence is available should carefully designed human observational or interventional studies be considered.

## Data Availability

The original contributions presented in this study are included in the article and Supplementary Materials. Further inquiries can be directed to the corresponding author.

## Supplementary Materials

The following supporting information can be downloaded at: https://www.mdpi.com/article/10.3390/dj14060331/s1, File S1: PRISMA_2020_checklist. Table S1: Full search strategies for each database.
