# Supplementary material for: Modulation of Orthodontic Tooth Movement by Statins: A Systematic Review of Animal Studies
Source: Dent J (Basel). 2026 Jun 1;14(6):331. doi: 10.3390/dj14060331 (PMC13298056; doi:10.3390/dj14060331)
Supplement: Supplementary file 1 [file dentistry-14-00331-s001.zip › Table S1 - Full search strategies for each database.pdf]

Table S1a – Summary of the main characteristics of the selected studies

First Author, year	Study design	Animal model	Sample Size	Pharmacological Administration	Orthodontic Device	Force (cN)	Force duration (days)	OTM (µm, SD)	Post-OTM Relapse [µm (SD)]	Histological Findings (TG compared to CG)	Molecular findings (TG compared to CG)	Outcome (TG compared to CG)
Han G, 2010[14]	prospective randomized trial	Wistar rats	32	2.5 mg/kg/die simvastatin; intraperitoneal injections	NiTi coil spring	50	21	NS	CG: 359µm (51)<br>TG: 195µm (32)	regular distribution of FB, smooth bone surfaces, less RP	increased OPG/RANKL ratio	reduced relapse, stimulation of PDL remodeling, increased bone formation
Vieira GM, 2015[15]	prospective split mouth trial on microCT images	Wistar rats	25	5mg/kg simvastatin; oral gavage	steel coil springs	75	18	NS	NS	NS	NS	no significant relapse inhibition
Dolci GS, 2017[16]	prospective randomized split mouth trial	Wistar rats	36	15mg/kg atorvastatine; oral gavage	NiTi coil spring	50	21	NS	CG: 31.91% (T1), 29.36% (T2), 28.40% (T3);^<br>TG: 9.59% (T1), 20.59% (T2), 7.94% (T3)^	reduction in OC count, increased cancellous bone/total bone volume ratio, transient OC inhibition	increased OPG	reduced relapse, increased OPG
AlSwafeeri H, 2018[17]	prospective randomized split mouth trial	White New Zealand Rabbits	10	0.5mg/480uL simvastatin solution; local iniections	NiTi coil spring	100	21	CG: 1530µm (340)<br>TG: 1550µm (360)	CG: 1150µm (390)<br>TG: 1010µm (540)	increased bone maturation, smooth bone surface, few resorption pits, regular distribution of fiber bundles	NS	no significant relapse inhibition, increased bone formation, reduced bone resorption
Dolci GS, 2018[18]	prospective randomized split mouth trial	Wistar rats	24	15mg/kg/day atorvastatine; gavage	NiTi coil spring	50	21	CG: 310.13µm (14.79) (T1) 381.38 (33.33) (T2) 485.85µm (68.94) (T3)°<br>TG: 263.53µm (14.79) (T1) 334.78µm (33.33) (T2) 439.25µm (68.94) (T3)°	NS	reduced OC count; bone-volume ratio similar to CG.	NS	temporary inhibition of osteoclastogenesis and OTM reduction

AlSwafeeri H, 2019[19]	prospective randomized split mouth trial	White New Zealand Rabbits	10	0.5mg/480uL simvastatin solution; local injections	NiTi coil spring	100	21	NS	difference in relapse (CG-TG): Comulative difference: 721µm (455) Weekly difference: 242µm (151)	less RP, less OC, more regular distribution of FB	NS	reduced relapse and bone resorption
Liu X, 2022[20]	prospective randomized trial	Sprague Dawley rats	32	2 mg /500µL simvastatin/ exososomes; local injections	NiTi coil spring	49.5*	14	NS	CG: 410µm (21.6)<br>TG: NS	Increase in amount and maturation of alveolar bone, absence of RP	increased *Runx2* and ALP	reduced relapse; increased osteogenesis

CG: control group
TG: test group
OC: osteoclasts
FB: fibroblasts
RP: resorption pits
PDL: periodontal ligament
NS: not specified

*grams (g) of force were converted in centiNewtons (cN) with the following equation: 1cN=1.01g
^T1: 7 days; T2: 14 days; T3: 21 days after appliance removal
°T1: 7 days; T2: 14 days; T3: 21 days from baseline

Table S1b - risk of bias and quality of the body evidence of the individual studies (SYRCLE's tool)

	Selection bias			Performance bias		Detection bias		Attrition bias	Reporting bias	Other
Article	Sequence generation	Baseline characteristics	Allocation concealment	Random housing	Blinding	Random outcome assessment	Blinding	Incomplete outcome data	Selective reporting	Other sources of bias
Han G, 2010[14]	Unclear	Low risk	Unclear	Unclear	Unclear	Unclear	Low risk	Low risk	Low risk	Low risk
Vieira GM, 2015[15]	High risk	Low risk	Unclear	Unclear	Unclear	Unclear	High risk	Low risk	High risk	Low risk
Dolci GS, 2017[16]	Unclear	Low risk	Unclear	Unclear	Unclear	Unclear	Low risk	Low risk	Low risk	Low risk
AlSwafeeri H, 2018[17]	Low risk	Low risk	Low risk	Low risk	Low risk	Low risk	Low risk	Low risk	Low risk	Low risk
Dolci GS, 2018[18]	Unclear	Low risk	High risk	Unclear	Unclear	Unclear	High risk	Low risk	Low risk	Low risk

AlSwafeeri H, 2019[19]	Low risk	Low risk	Low risk	Unclear	Low risk	Low risk	Low risk	Low risk	Low risk	Low risk
Liu X 2022[20]	Unclear	Low risk	Unclear	Unclear	Unclear	Unclear	Low risk	Low risk	Low risk	Low risk
